# Novel therapeutic evaluation biomarkers of lipid metabolism targets in uncomplicated pulmonary tuberculosis patients

**DOI:** 10.1038/s41392-020-00427-w

**Published:** 2021-01-18

**Authors:** Jia-Xi Chen, Yu-Shuai Han, Shan-Qiang Zhang, Zhi-Bin Li, Jing Chen, Wen-Jing Yi, Huai Huang, Ting-Ting Jiang, Ji-Cheng Li

**Affiliations:** 1 Institute of Cell Biology and Department of Cardiology of the Second Affiliated Hospital, Zhejiang University School of Medicine, 310058 Hangzhou, China; 2 The Medical Research Center of Yue Bei People’s Hospital, Shantou University Medical College, 512025 Shaoguan, China; 3 Department of Histology and Embryology, Shaoguan University School of Medicine, 512025 Shaoguan, China; 4 Taizhou Hospital of Zhejiang Province affiliated to Wenzhou Medical University, 318050 Taizhou, China

**Keywords:** Predictive markers, Respiratory tract diseases, Molecular medicine

## Abstract

Currently, the management of pulmonary tuberculosis (TB) lacks potent medications and accurate efficacy evaluation biomarkers. In view of the fact that the host lipids are the important energy source of *Mycobacterium tuberculosis* (*Mtb*), UPLC-MS/MS based on lipid metabolism was used to monitor the plasma lipid spectrum of TB patients from the initial diagnosis to cured. The analysis showed that TB patients presented aberrant metabolism of phospholipids, glycerides, and sphingolipids. Upon the treatment, the abnormal expression of Cer (d18:1/24:0), CerP (d18:1/20:3), LPE (0:0/22:0), LPA (0:0/16:0), and LPA (0:0/18:0) in TB patients were gradually normalized, indicating that the intervention of lipid metabolism could block energy metabolism and inhibit the cell wall synthesis of *Mtb*. Furthermore, the increase in ceramide (Cer) levels could promote autophagosome–lysosome fusion. LPA (0:0/16:0) and LPA (0:0/18:0) had a great potential in the early diagnosis (both sensitivity and specificity were 100%) and efficacy evaluation (both sensitivity and specificity were 100%) of TB, indicating that the above lipid metabolites could be used as potential biomarkers for TB.

## Abstract (web summary)

Lipidomics profiling workflow. **(a)** Plasma lipidomics extraction was accomplished by fractionating samples into pools of species with similar physicochemical properties using appropriate combinations of organic solvents, after the addition of an internal standard. **(b)** UPLC-MS/MS-based platforms were used to perform optimal profiling of plasma lipidomics. Data preprocessing generated a list of chromatographic peak areas for the lipidomics detected in each sample injection. Inter- and intra-batch normalization was carried out with quality control (QC) calibration and internal standard correction. **(c)** Once normalized, univariate and multivariate data analyses were performed. **(d)** Selection of the best candidate biomarkers for the early diagnosis and efficacy evaluation of TB. **(e)** The results of this experiment provide four ideas for the research and development of potential antituberculotic medications, so as to achieve the purpose of antituberculosis treatment.

## Graphical abstract

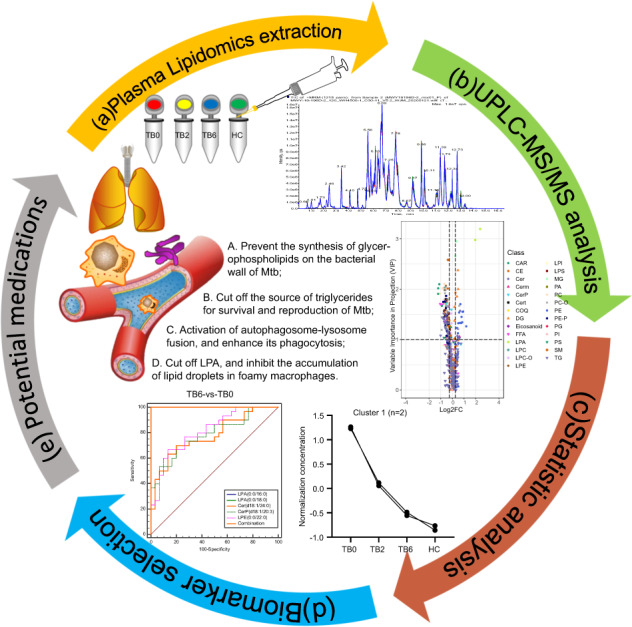

## Introduction

Pulmonary tuberculosis (TB) is a chronic pulmonary infectious disease caused by *Mycobacterium tuberculosis* (*Mtb*), which has affected humans for nearly 70,000 years.^1^ Although the cure rate of TB has been significantly improved after the implementation of directly treatment short-course chemotherapy (DOTS) approved by the WHO, the global data of TB treatment outcome indicate that the success rate of 6-month standard treatment for emerging TB is only 85%.^2^ The global tuberculosis recurrence rate varies from 2.3% to 6.5%,^3–5^ while the recurrence rate in China is 11.8%.^6^ In addition, 18% of treated patients developed multidrug-resistant TB (MDR-TB).^2^ Due to the low specificity of sputum smear, the cure of TB currently is only determined based on clinical symptoms, computed chest tomography (CT), and drug treatment course. For patients diagnosed with pulmonary TB, routine treatment will last for at least 6 months, including multiple times of CT examinations during the whole course of treatment. However, the imaging evidence is nonspecific, and many pulmonary pathologic changes can be reflected on CT. Currently, there is a lack of laboratory standards for evaluating the efficacy of TB treatment, hence it is a need to study the biomarkers for accurately evaluating the therapeutic effect of TB.

The host plasma is rich in lipids, which is the major nutrition source for the growth and reproduction of *Mtb*. *Mtb* infection can induce accumulation of cholesteryl ester and glyceride in macrophages, leading to the formation of foamy macrophages and tuberculous granuloma.^7^ The fatty acid composition of triglyceride (TAG) in *Mtb* is almost the same as that in the host. The bacteria can use their own TAG synthases together with the host TAG to make lipid droplets.^8^ Furthermore, *Mtb* preferentially migrates to lipid droplets in the host foamy macrophages and engulf lipid droplets as a source of long-term nutrition.^9^ However, the effect of antituberculosis medications on lipid metabolism of the host has not been reported up to now. In the previous proteomic studies, we found that the expression of lipid metabolism-related lipoproteins (a) was upregulated in the cured TB patients, but the changes of the downstream lipids are still unclear.^10^ Therefore, it is of great value to investigate the changes of host plasma lipids in TB patients upon the antituberculosis treatment.

In this study, the high-throughput detection of alterations in the whole lipid metabolome of the host caused by *Mtb* infection was carried out by using ultra-performance liquid chromatography-tandem mass spectrometry (UPLC-MS/MS) technology. The changes in host plasma lipid metabolism were analyzed under the antituberculosis treatment, and the biomarkers for evaluating the therapeutic efficacy of TB patients were screened to reveal the potential lipid metabolism targets for the treatment of TB.

## Results

### Raw mass spectrometry data preprocessing

There was no statistical difference in the mean age, gender, and BMI between TB0, TB2, TB6, and HC groups (*P* > 0.05). The positive rates of smear, culture, PCR chip, Xpert MTB/RIF, and T-SPOT in TB patients are shown in Table 1, no significant difference was found among these groups (*P* > 0.05).

**Table 1:** Statistics of clinical characteristics and laboratory indexes of the study cohort

	Healthy controls	Untreated TB	2 Months	6 Months	
	(*N* = 30)	(*N* = 30)	(*N* = 30)	(*N* = 30)	*P* value
Age (median, IQR)	33.5 (27.7–47.7)	32.0 (22.7–54.0)	32.0 (25.7–45.7)	34.0 (25.2–47.5)	0.924^a^
BMI (median, IQR)	21.1 (20.0–22.3)	20.2 (16.4–24.0)	20.9 (19.0–21.6)	20.9 (18.2–24.4)	0.849^a^
Gender (male)	17 (56%)	13 (43%)	18 (60%)	20 (67%)	0.317^b^
Sputum smear: positive, no. (%)	/	16 (53%)	14 (47%)	14 (47%)	0.837^b^
Xpert MTB/RIF: positive, no. (%)	/	20 (67%)	19 (63%)	19 (63%)	0.953^b^
PCR chip: positive, no. (%)	/	10 (33%)	12 (40%)	16 (53%)	0.279^b^
Cultivate: positive, no. (%)	/	16 (53%)	16 (53%)	16 (53%)	1.000^b^
T-spot.TB: positive, no. (%)	/	20 (67%)	19 (63%)	20 (67%)	0.952^b^

*N* number of subjects, *TB0* untreated TB patients, *TB2* 2-month-treated TB patients, *TB6* cured TB subjects, *HC* health controls

^a^*P* value among four groups from Kruskal–Wallis H test

^b^*P* value among four groups from the Chi-square test

ESI-QTRAP-MS/MS technology was used to perform a targeted detection of a wide range of plasma lipid metabolites in TB0, TB2, TB6, and HC groups. After baseline filtering, peak recognition, integration, retention time correction, peak alignment, and normalization, 23 types of lipid metabolites and 537 lipid substances were obtained. The QC results showed that the positive and negative TIC ion current patterns presented good reproducibility, in which 448 peaks were detected in the positive ion pattern and 89 peaks detected in the negative ion pattern (Supplementary Fig. 1).

### Plasma lipid metabolite profile of active TB

**Fig. 1:**
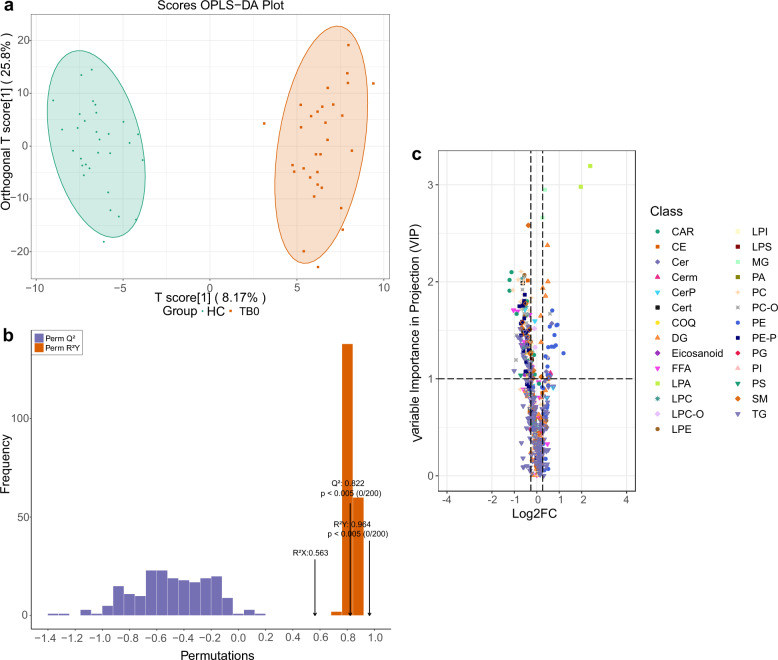
Establishment and validation of OPLS-DA model (TB0 group vs. HC group), and screening of differential lipids. **a** The OPLS-DA model was able to separate the TB0 group from HC group clearly based on the selected lipid metabolites. **b** The model had good prediction ability, permutation test showed that R2X (cum) = 0.563, R2Y(cum) = 0.964, Q2(cum) = 0.822. **c** The differential lipids between the two groups were identified by volcanic map. The abscissa showed the different fold changes (logFC) of each lipid substance in different groups, the ordinate showed VIP value, and the different scatter colors and shapes represented the screening results of different lipid categories

OPLS-DA model showed that the lipid metabolites in the TB0 group and the HC group were clearly separated (Fig. 1a). It can be seen that there was a significant plasma lipid metabolism change in the plasma of patients with TB. Variable importance in the projection (VIP) of each different lipid variable was obtained by modeling, as this factor explains to which extent a variable contributes in the projection and the metabolites obtained a VIP > 1.0 could be considered as the most relevant contributors to explain the differences. Under the condition of VIP value ≥1.0, the main types of differential plasma lipid metabolites between the TB0 group and HC group were phospholipids, glycerides, cholesterol lipids, ceramide, acylcarnitine, etc. The scatter plots of the OPLS-DA model can reliably distinguish the TB0 group from the HC group (Fig. 1b). The resulting R^2^X, R^2^Y, and Q^2^ of permutation test model validation were 0.563, 0.964, and 0.822, respectively (*P* < 0.005). By the cutoff of fold change >1.2 or <1/1.2, and VIP ≥ 1.0, 163 differential lipids were identified in patients with TB compared to HC, of which 134 were downregulated and 29 were upregulated (Fig. 1c). The downregulated lipids were mainly in the families of sphingomyelin (SM), phosphatidylcholine (PC), lysophosphatidylethanolamine (LPE), ceramide (Cer), while the upregulated lipids were lysophosphatidic acid (LPA), triglyceride (DG), monoglyceride (MG), etc. We further evaluated the statistical significance of different lipids by *t* test combined with ROC, 30 metabolites with the higher area under the curve (AUC) value were obtained from the comparison between the TB0 and the HC groups, which could be used to screen TB from the general healthy population, with a better screening efficiency (Table 2). Among them, two lysophosphatidic acids (LPA) increased significantly, the AUC area of LPA (0:0/16:0) and LPA (0:0/18:0) were 1 (95% CI, 1.000–1.000), sensitivity and specificity were 100 and 100%, respectively. Furthermore, the AUC area of monoglyceride (MG), a degradation product of glyceride, was 0.984, the sensitivity and specificity were 100% and 93.3%, respectively.

**Table 2:** Statistics of 30 lipid metabolites distinguish TB0 from HC

Metabolite	AUC	Log2FC	Type
LPA (0:0/16:0)	1	1.963	Up
LPA (0:0/18:0)	1	2.382	Up
MG (18:0/0:0/0:0)	0.984	0.367	Up
SM (d18:0/12:0)	0.953	−0.372	Down
SM (d18:2/16:1)	0.818	−0.489	Down
DG (18:0/18:0/0:0)	0.892	0.478	Up
DG (16:0/20:0/0:0)	0.844	0.497	Up
DG (16:0/18:0/0:0)	0.818	0.267	Up
DG (14:0/22:0/0:0)	0.793	0.391	Up
Decenoyl-carnitine	0.861	−1.126	Down
PC (O-16:2/18:1)	0.844	−0.541	Down
PC (18:1/22:6)	0.84	−0.697	Down
PC (18:2/22:6)	0.84	−0.605	Down
PC (20:1/22:6)	0.833	−0.831	Down
PC (14:0/22:6)	0.83	−1.062	Down
PC (16:0/22:6)	0.827	−0.596	Down
PC (16:1/18:2)	0.81	−0.575	Down
PC (O-20:4/20:4)	0.806	−0.638	Down
PC (16:1/22:6)	0.799	−0.731	Down
PC (18:2/18:2)	0.798	−0.678	Down
PC (O-16:0/14:0)	0.797	−0.561	Down
PC (16:0/18:2)	0.793	−0.304	Down
Octanoyl-carnitine	0.838	−1.214	Down
CE (18:2)	0.834	−0.401	Down
LPC (22:0/0:0)	0.832	−0.641	Down
LPE (0:0/22:0)	0.832	−0.549	Down
Cer (d18:0/24:0)	0.821	−0.349	Down
Decanoyl-carnitine	0.816	−1.216	Down
PE (P-20:1/20:0)	0.812	−0.533	Down
FFA (22:6)	0.789	−0.859	Down

*AUC* area under lipid curve

### Changes of plasma lipid profile after antituberculosis treatment

**Fig. 2:**
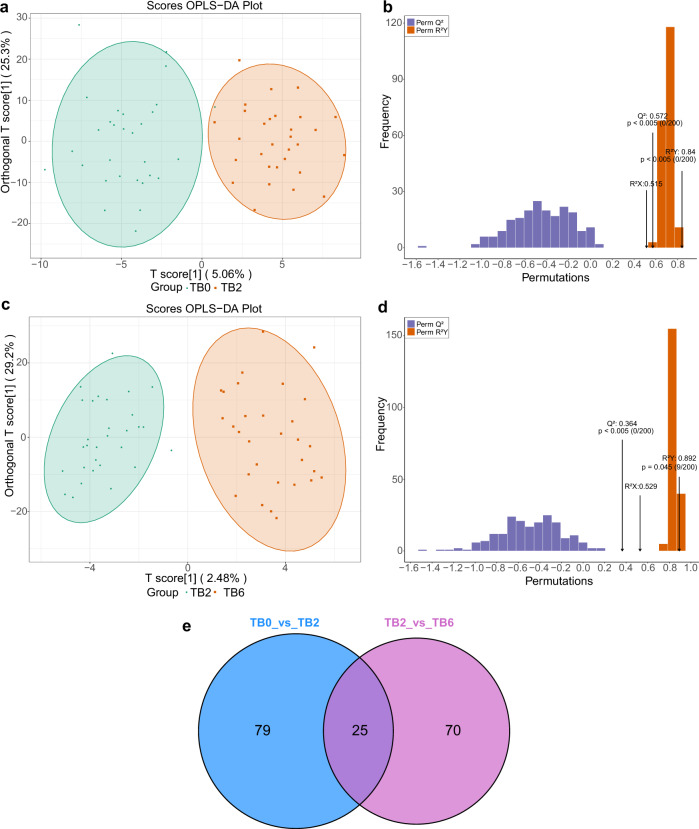
Establishment and validation of OPLS-DA model (TB0 group vs. TB2 group, TB2 group vs. TB6 group), and screening of dynamically changing lipids. **a** OPLS-DA model was able to separate the TB2 group from the TB0 group clearly based on the selected lipid metabolites. **b** The model has good prediction ability, permutation test showed that R2X (cum) = 0.515, R2Y(cum) = 0.840, Q2(cum) = 0.572. **c** OPLS-DA model was able to separate the TB6 group from the TB2 group clearly based on the selected lipid metabolites. **d** The model has good prediction ability, permutation test showed that R2X (cum) = 0.529, R2Y(cum) = 0.892, Q2(cum) = 0.364. **e** In all, 25 lipid metabolites with the significant statistical difference between the TB0 group and TB2 group, and TB2 group and TB6 group were found in the Venn intersection of the comparison

The TB2 and TB0 groups can be distinguished by scatter plots of the OPLS-DA model (Fig. 2a). The verification results of the permutation test to the model showed that R^2^X was 0.515, R^2^Y was 0.840, and Q^2^ was 0.572 (*P* < 0.005) (Fig. 2b). The TB6 and TB2 groups can also be distinguished by scatter plots of the OPLS-DA model (Fig. 2c). The permutation test of the model showed that R^2^X was 0.529, R^2^Y was 0.892, and Q^2^ was 0.364(*P* < 0.005) (Fig. 2d). Furthermore, 25 lipid metabolites with a significant statistical difference were commonly identified as the differential metabolites by the comparison both between the TB0 group and TB2 group, and TB2 group and TB6 group (Fig. 2e).

**Fig. 3:**
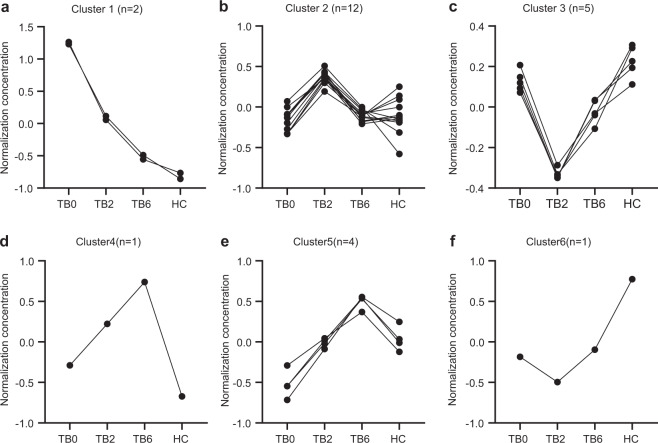
K-means cluster showing 25 differential lipids among TB0, TB2, TB6, and HC groups. **a** The ordinate is the normalized lipid concentration. The abscissa is the TB0, TB2, TB6, and HC groups. Cluster 1 had a downward trend along with the TB0, TB2, and TB6 groups. **b** The lipid groups of cluster 2 showed an upward trend in the TB2 group, but similar low values in Tb0, TB6, and HC groups. **c** Cluster 3 had a downward trend in the TB2 group and an upward trend in the TB6 group. **d** Cluster 4 showed a single-lipid cluster, which was low in TB0 and HC groups but increased in TB2 and TB6 groups with antituberculosis treatment. **e** Cluster 5 had an upward trend along with the TB0, TB2, and TB6 groups. **f** Cluster 6 showed a single-lipid cluster, which was a high level in the HC group and low level in TB patients, especially in the TB2 group

After 2-month intensive-phase treatment (TB2) and 6-month intensive plus continuation phase treatment (TB6), there was a significant change in the abundance of these 25 lipids (Table 3). K-means cluster analysis was employed to classify these lipid substances with the same change trend and resulted in six clusters with different expression patterns across different patient groups. In cluster 1, lysophosphatidic acid LPA (0:0/16:0) (TB0/HC ratio = 3.99) and LPA (0:0/18:0) (TB0/HC ratio = 5.49) were significantly upregulated in untreated TB0 patients, but shown a significant downward trend after treatment, and the plasma abundance of LPA (0:0/16:0) (TB6/HC ratio = 1.33) and LPA (0:0/18:0)(TB6/HC ratio = 1.82) in the TB6 group was nearly restored to the normal level found in the HC group (Fig. 3a). There were 12 lipids classified in cluster 2, which presented low levels in the TB0, TB6, and HC groups, but higher levels in the TB2 group (Fig. 3b). This changing pattern in lipid levels may be related to intensive treatment and was found as well in cluster 6, a single-lipid aggregation (Fig. 3f). Furthermore, a single lipid classified in cluster 4 showed a low level in the TB0 and HC groups, which gradually increased in TB2 and TB6 groups (Fig. 3d). In cluster 3, triglycerides were mainly accumulated, including TG (16:0/16:0/22:6)(TB2/HC ratio = 0.36), TG (16:0/16:1/22:5) (TB2/HC ratio = 0.64), TG (14:0/20:2/20:5) (TB2/HC ratio = 0.53), and TG (16:0/16:1/22:6) (TB2/HC ratio = 0.6 ratio = 0.53), which decreased significantly during the 2-month intensive-phase treatment. After the intensive and continuation phase treatment for 6 months, their expression levels changed significantly, with the levels of TG(16:0/16:0/22:6) (TB6/HC ratio = 0.66), TG (16:0/16:1/22:5) (TB6/HC ratio = 0.85), TG (14:0/ 20:2/20:5) (TB6/HC ratio = 0.83), and TG (16:0/16:1/22:6) (TB6/HC ratio=0.85) nearly normalized. These results indicated that the change of triglyceride can interfere with the energy supply of *Mtb* in the course of intensive treatment (Fig. 3c). Furthermore, in cluster 5, we found that Cer (d18:1/24:0)(TB0/HC ratio = 0.79), CerP (18:1/20:3) (TB0/HC ratio = 0.76), LPE (0:0/22:0) (TB0/HC ratio = 0.69), and TG (14:1/14:1/22:3)(TB0/HC ratio = 0.86) were significantly downregulated in the plasma of untreated patients in TB0, but first gradually upregulated in the patients received intensive treatment and then roughly normalized at the end of 2-month intensive-phase treatment. The plasma abundance of these four metabolites was completely normalized in the cured patients who completed the 6-month treatment (Fig. 3e).

**Table 3:** 25 Differential lipid metabolites among TB0, TB2, TB6, and HC

			Ratios					
No.	Differential compounds	Ion mode	TB0/HC	TB2/HC	TB6/HC	TB0/TB2	TB0/TB6	TB2/TB6
1	LPA (0:0/16:0)	N	3.99	2.16	1.33	1.85	3	1.62
2	LPA (0:0/18:0)	N	5.49	2.99	1.82	1.84	3.02	1.64
3	PC (20:3/18:1)	N	1.39	1.79	1.36	0.78	1.03	1.32
4	PC (16:0/20:5)	N	0.92	0.6	0.74	1.53	1.24	0.81
5	PC (18:2/20:3)	N	1.13	1.5	1.18	0.75	0.96	1.28
6	Myristoyl-carnitine	P	0.77	1.07	0.89	0.72	0.87	1.21
7	Dodecenoyl-carnitine	P	0.66	0.97	0.79	0.68	0.84	1.24
8	Tetradecenoyl-carnitine	P	0.77	1.23	0.93	0.63	0.83	1.33
9	Palmitodileoyl-carnitine	P	0.68	1.18	0.87	0.57	0.78	1.36
10	Cer (d18:1/20:0)	P	1.36	1.84	2.31	0.74	0.59	0.8
11	Cer (d18:1/24:0)	P	0.79	1.03	1.27	0.77	0.62	0.81
12	CerP (d18:1/20:3)	P	0.76	0.97	1.23	0.78	0.62	0.79
13	LPE (0:0/22:0)	P	0.69	0.88	1.09	0.78	0.63	0.81
14	PE (P-20:2/22:3)	P	0.67	0.54	0.7	1.24	0.96	0.77
15	TG (18:1/18:3/20:0)	P	1.09	1.31	0.95	0.83	1.15	1.38
16	TG (16:1/20:1/20:2)	P	1.05	1.34	1.03	0.78	1.02	1.31
17	TG (18:2/18:2/20:0)	P	1	1.27	0.96	0.78	1.04	1.32
18	TG (14:1/14:1/22:3)	P	0.86	1.12	1.34	0.77	0.64	0.84
19	TG (18:2/18:3/20:0)	P	0.98	1.36	0.97	0.72	1.02	1.41
20	TG (18:0/18:3/20:2)	P	1.06	1.37	1.03	0.77	1.03	1.34
21	TG (18:1/18:3/20:1)	P	0.97	1.36	1.07	0.71	0.91	1.28
22	TG (16:0/16:0/22:6)	P	0.77	0.36	0.66	2.17	1.17	0.54
23	TG (16:0/16:1/22:5)	P	1.08	0.64	0.85	1.69	1.27	0.75
24	TG (14:0/20:2/20:5)	P	0.9	0.53	0.83	1.69	1.08	0.64
25	TG (16:0/16:1/22:6)	P	0.94	0.53	0.85	1.77	1.1	0.62

*P* positive, *N* negative

### KEGG pathway analysis and changes of enrichment pathway under antituberculosis treatment

**Fig. 4:**
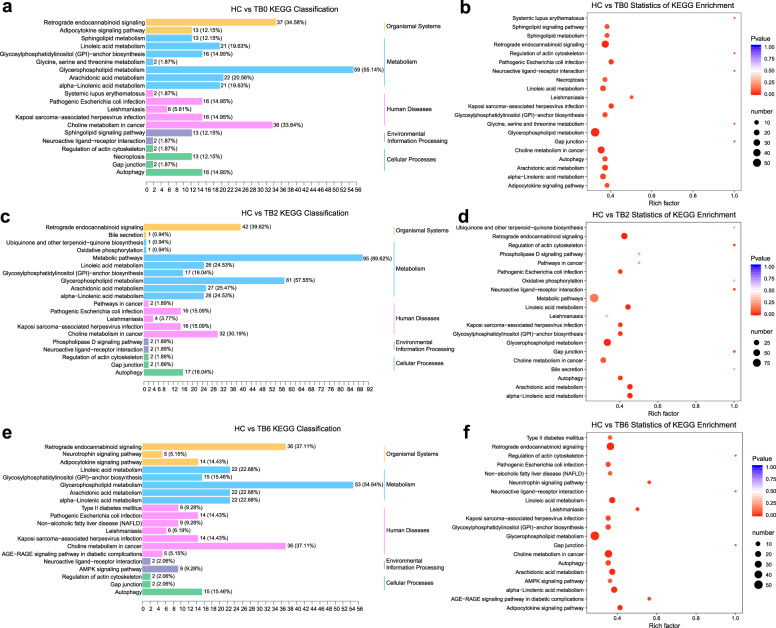
The lipidome KEGG enrichment analysis of TB patients. **a**, **b** KEGG pathway analysis of TB0 unique to the HC groups. The color of bubbles represents the value of adjusted *P* value, and the size of bubbles represents the number of counts. **c**, **d** KEGG pathway analysis of TB2 unique to the HC groups. **e**, **f** KEGG pathway analysis of TB6 unique to the HC groups

KEGG pathway enrichment analysis of all differential lipid metabolites found that the plasma between the TB0 and HC groups was different in the metabolism pathways of glycerol phospholipid, sphingolipid metabolism, and autophagy (*P* < 0.05) (Fig. 4a, b). We have found that autophagosome maturation plays a key role in the occurrence of active tuberculosis in the previous study, so we focused on the autophagy signaling pathway. Compared to the HC group, there were significant changes in the levels of 40 phosphatidylcholines (PC) in the TB0 group, 38 of which were downregulated, accounting for 41.3% (38/92) of the total detected PC. Along the course of antituberculosis treatment, the proportion of downregulated PC decreased gradually, which was 27.2% (25/ 92) in the TB2 group and 22.8% (21/92) in the TB6 group, and 11.9% of PC (11/92) was upregulated in TB6 group. In addition, sphingomyelin (SM) was downregulated in TB patients before treatment, accounting for 34.6% (9/26) of the total metabolites. The fraction of the downregulated SM accounted for 11.5% (3/26) after TB was cured. During the antituberculosis treatment, the downregulation of phospholipids was mitigated, which might be used as the indicator for the effect of antituberculosis treatment. Compared with the HC group, 16 lipids involved in the autophagy signaling pathways presented significant changes in the TB0 group, in which 15 were upregulated and accounted for 34.9% (15/43) of the total detected lipids in the autophagic signaling pathway, and another one was downregulated. In subsequent treatment, we also found that all of 17 lipids in the autophagic signaling pathway presented significant upregulation in the TB2 group accounting for 39.5% (17/43) of the total detected lipids in the autophagic signaling pathway. In the TB6 group relative to TB2 group, there was one more upregulated lipid related to the autophagic signaling pathway. Furthermore, in the enriched pathways of differential lipids between the TB2 group and the HC group, and between the TB6 group and the HC group, the enrichment of linolenic acid and arachidonic acid metabolism pathways increased with the progression of treatment (Fig. 4c, d–f).

### The lipid biomarkers for the potential therapeutic evaluation of active TB

**Fig. 5:**
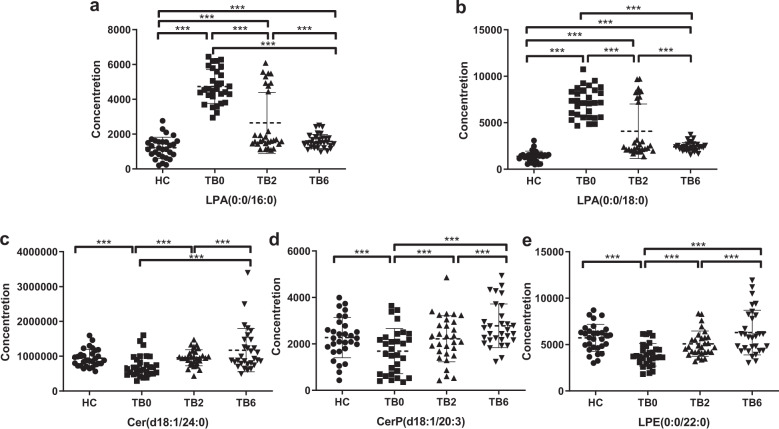
The changes of differential lipids in HC, TB0, TB2, and TB6 groups. **a** Relative concentration of LPA (0:0/16:0) in HC, TB0, TB2, and TB6 groups. **b** Relative concentration of LPA (0:0/18:0) in HC, TB0, TB2, and TB6 groups. **c** Relative concentration of Cer (d18:1/24:0) in HC, TB0, TB2, and TB6 groups. **d** Relative concentration of CerP (d18:1) in HC, TB0, TB2, and TB6 groups. **e** Relative concentration of LPE(0:0/22:0) in HC, TB0, TB2, and TB6 groups. Mann–Whitney *U* test was used to test the statistical significance. ***P < 0.001

The results of the nonparametric test among groups showed that the plasma abundance of five lipid metabolites in patients with TB presented significant statistical difference (*P* < 0.001), including LPA (0:0/16:0), LPA (0:0/18:0), Cer (d18:1/24:0), CerP (d18:1/20:3), and LPE (0:0/22:0). These metabolites were closely related to *Mtb* infection and the treatment status of the host, in which LPA (0:0/16:0) and LPA (0:0/18:0) were significantly increased when TB patients were untreated. It can be seen from the scatter plots that in the TB2 group, the plasma LPA level in most of the patients was significantly reduced to a lower level, while the LPA level of the other nine patients was still as high as that in untreated patients. The pulmonary imaging of these nine patients were reviewed which manifested that the absorption of the lesions was unfavorable (Fig. 5a, b). In addition, the levels of Cer (d18:1/24:0), CerP (d18:1/20:3), and LPE (0:0/22:0) were significantly low when TB was untreated and gradually raised to the level in the HC group with the progression of treatment (Fig. 5c–e).

### Evaluation of the therapeutic effect of lipid markers on active TB

**Fig. 6:**
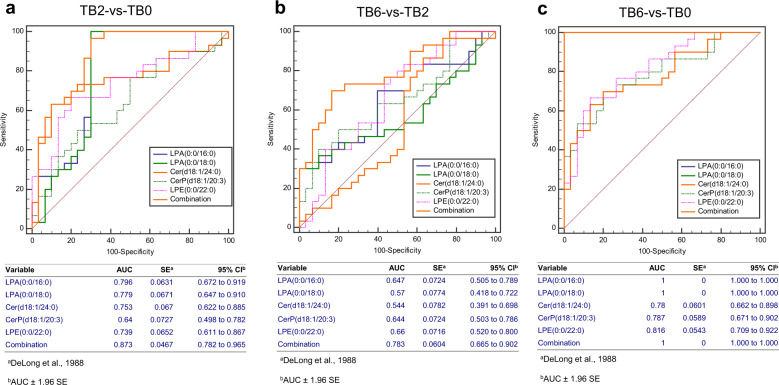
Evaluation of five differential lipid metabolites on the therapeutic effect of active TB treatment. **a** ROC curve was used to distinguish the patients in the TB2 and TB0 groups, the area of the panel under the curve (AUC) was 0.873 (95% CI, 0.782–0.965). **b** ROC curve was used to distinguish patients in the TB6 and TB2 groups, the area of the panel under the curve (AUC) was 0.783 (95% CI, 0.665–0.902). **c** In addition, the ROC curve was used to distinguish patients in the TB6 and TB0 groups, the area of the panel under the curve (AUC) was 1.000 (95% CI, 1.000–1.000)

*T* test combined with ROC curve was used to evaluate the ability of five differential lipids to assess the therapeutic effect in the patients in the TB2 and TB6 groups, including Cer (d18:1/24:0), CerP (d18:1/20:3), LPE (0:0/22:0), LPA (0:0/16:0), and LPA (0:0/18:0). The panel of these five differential lipids can be used to distinguish the TB2 patients from the TB0 group patients, with an area under the curve (AUC) of 0.873 (95% CI, 0.782–0.965) (Fig. 6a), as well as the TB6 patients from the TB2 group patients, with an area under the curve (AUC) of 0.783 (95% CI, 0.665–0.902) (Fig. 6b). Furthermore, the panel of the five differential lipids can robustly distinguish the TB6 patients from the TB0 group patients, and the area under the curve (AUC) was 1.000 (95% CI, 1.000–1.000) (Fig. 6c). Among them, two lysophosphatidic acids demonstrated the potent ability to distinguish between cured and uncured active TB patients, as evidenced by both AUC area of LPA (0:0/16:0) and LPA (0:0/18:0) of 1 (95% CI, 1.000–1.000), and the sensitivity and specificity of both 100%.

## Discussion

The main pathological features of TB are foamy macrophages, tuberculous granuloma, and caseous necrosis.^11^ Abundant cholesterylesters and glycerides accumulate in lipid droplets in foamy macrophages isolated from *Mtb*-infected granulomas.^7^ The genome sequencing of *Mtb* H37Rv and CDC1551 has revealed that *Mtb* has more than 250 genes related to lipid metabolism.^12,13^ The transcription of some genes can be induced by *Mtb* infection. And some genes encode molecules necessary for *Mtb* growth and reproduction such as lipase,^14^ isocitrate lyase,^15^ phospholipase,^16^ mycotic acid lyase,^17^ free fatty acid coenzyme A (CoA),^18^ and acyl-coenzyme A dehydrogenase.^19^ The mutation of genes related to lipid metabolism can alleviate the symptoms of *Mtb*-infected cells or animals.^20–22^

In this study, large-scale targeted lipidomics with UPLC-MS/MS was used to screen the dynamic spectrum of lipid metabolism changes in the plasma of patients with TB during the treatment period. It was found that *Mtb* infection could cause abnormal expression of various lipid metabolites in the signaling pathways of glycerides, phospholipids, and sphingolipids in the plasma of the TB patients, indicating that *Mtb* uses host glycerophospholipids to maintain its survival, reproduction, metabolism, and cell wall structure.^8,23^ In the previous study of untargeted metabolomics, we found that arachidonic acid AA and the metabolite of glycerophospholipids decreased significantly in the TB0 group and then increased significantly and further normalized along the TB2 to TB6 groups,^24^ which was consistent with the results of this targeted lipidomics study. Thromboxane B2 (TXB2), the metabolite of arachidonic acid AA, also decreased significantly in untreated TB patients and increased significantly with the progression of treatment, and finally returned to the normal level. Previous studies have demonstrated that the level of plasma lysophosphatidylcholine (LPC) in patients with TB significantly decreased.^25,26^ Consistent with these results, we also found that a variety of phosphatidylcholine (PC), lysophosphatidylcholine (LPC), and lysophosphatidylethanolamine (LPE) decreased significantly in the plasma of untreated patients with TB, while restored to the normal level in patients achieved cure after intensive treatment.

In addition, we report for the first time that lysophosphatidic acid (LPA), a metabolite of glycerophospholipids, has an abnormally high abundance in the plasma of untreated patients with TB. The sensitivity of LPA for early biological diagnosis of TB was 100%. With the progression of treatment, after 2 months of intensive-phase treatment and 6 months of intensive plus continuation treatment, the abundance of LPA significantly decreased, and their sensitivity and specificity for the evaluation of cured TB were both 100%, indicating that they are ideal biomarkers for evaluating the therapeutic effect in TB patients. LPA is an effective bioactive phospholipid, which can induce cell proliferation, migration, cytokine release, and other cellular reactions. It can be produced within and outside of cells through the deacetylation of phosphatidic acid, the phosphorylation of monoacylglycerol, and the hydrolysis of lysophosphatidylcholine.^27^ Delogu et al.^28^ found that LPA may play an important protective role in the primary infection of *Mtb* by enhancing the innate immune response of macrophages and alveolar epithelial cells. Furthermore, Tsukahara and others^29^ and Zhang et al.^30^ all reported that lysophosphatidic acid (LPA) can activate intracellular transcription factor peroxisome proliferator-activated receptorγ (PPAR-γ), while PPAR-γ activation during *Mtb* infection can induce accumulation of lipid droplets in foamy macrophages,^31,32^ antagonize or stimulate upstream regulator (such as vitamin D receptor) to inhibit the activation of PPAR-γ.^33^ Elimination of the accumulation of lipids in macrophages infected with *Mtb* can inhibit the growth of *Mtb* in the host. Therefore, we speculated that the inhibition of LPA production during *Mtb* infection can inhibit the PPAR-γ gamma signaling pathway, thereby further inhibiting the formation of foamy macrophages, tuberculous granuloma, and caseous necrosis caused by *Mtb* infection, and specifically inhibiting phospholipase may be a therapeutic strategy to inhibit the reproduction and spread of *Mtb*.

This study found that the level of certain types of triglycerides and free fatty acids decreased simultaneously in TB, while was restored when TB was cured. Triglycerides are an important component in the caseous necrosis tissue of TB. As the storage of endogenous energy and free fatty acids, triglycerides can help *Mtb* recover from hypoxia, hunger, and other environmental stresses, which is related to the virulence of *Mtb.*^34^ The lipase of *Mtb* can decompose triglycerides into diacylglycerides (DG) and monoacylglycerides (MG). The released medium-chain fatty acids are synthesized into long-chain fatty acids through Fas I/II,^35^ forming the main components of lipids in the *Mtb* wall, such as mycobacterial acid and methyl branched-chain fatty acids. Isoniazid, the first-line drug of antituberculosis, can prevent the synthesis of mycotic acid by targeting the fatty acid elongation of fatty acid synthetase II complex, so as to inhibit *Mtb* growth.^36^

Other studies have found that lipid function is not limited to the energy storage of membrane structural components or cells, but also includes a series of signal transduction, phagosome formation, and maturation functions.^37,38^ In the previous studies, we found that miR-423-5p in the serum of patients with TB increased significantly, and the inhibition of autophagosome maturation by inhibiting autophagosome–lysosome fusion played a key role in the pathogenesis of TB.^39^ Specific bioactive lipids such as sphingomyelin and ceramide can activate the assembly and maturation of actin in phagocytes, thus triggering the fusion of phagosomes and lysosomes to kill pathogenic mycobacteria in macrophages.^40,41^ In our study, it was found that various sphingomyelin and ceramide in plasma sphingomyelin metabolic pathways were significantly reduced during the phase of *Mtb* reproduction and bacterial spread, and these lipid metabolites returned to the normal levels after 2-month intensive-phase treatment and the achievement of cure. KEGG signaling pathway showed that with the progression of treatment, autophagy signaling pathway-related lipids in patients with TB showed a trend of gradual upregulation. The decrease of plasma sphingomyelin and ceramide in patients with TB reflected the imbalance of sphingolipid metabolism during *Mtb* infection. The significant decrease of sphingomyelin and ceramide in TB may inhibit the fusion of phagosome and lysosome, leading to the spread of *Mtb* and the immune escape. After the treatment of 2 months and the whole course, the levels of sphingomyelin and ceramide were gradually normalized, indicating the restoration of the autophagy pathway in the host with cured TB. Taking all these together, we speculate that medications targeting these bioactive lipids in macrophages infected with *Mtb*, or to improve the level of ceramide in the circulating blood of the host, so as to regulate autophagosome–lysosome fusion will enhance largely the antibacterial response of the host.

In conclusion, the results of this experiment provide four ideas for the research and development of antituberculosis medications, so as to for achieving the purpose of antituberculosis treatment. (1) interfering with the expression of *Mtb* phospholipase, inhibiting the host’s uptake of glycerophospholipids, preventing the synthesis of lipid on the bacterial wall of *Mtb* to lead to bacterial death. (2) interfering with the expression of *Mtb* lipase, inhibiting the catabolism of triglycerides, blocking the source of energy for survival and reproduction of *Mtb* to obtain “starving bacteria”. (3) targeting the delivery of ceramide to macrophages infected with *Mtb*, activation of autophagosome–lysosome fusion, and enhancing its phagocytosis. (4) blocking the source of glycerol phospholipid metabolite LPA, preventing transcription factor PPAR-γ activation, and inhibiting the accumulation of lipid droplets in foamy macrophages. Meanwhile, LPA (0:0/16:0) and LPA (0:0/18:0) can be used as potential biomarkers for the evaluation of the therapeutic effect of TB.

## Materials and methods

### Study cohort

From May to December 2019, plasma samples of 30 newly diagnosed TB patients before starting treatment (TB0 group), 30 TB patients after 2-month intensive-phase treatment (TB2 group), 30 cured TB patients after 6 months of intensive plus continuation phase treatment (TB6 group), and 30 healthy controls (HC group) were collected from the hospital of Taizhou Enze Medical Center (Group). The diagnosis of TB infection was made according to the following criteria: (A) Positive sputum examinations (smear or culture); (B) positive nucleic acid test of *Mtb*; (C) chest images (X-ray or CT scan) findings consistent with the active TB; (D) pathology diagnosis of TB in lung specimens; (E) response to antituberculous treatment. The enrolled patients were administrated with the standard TB therapeutic regimens and grouped according to the therapeutic outcome: TB2 group was treated with intensive treatment for 2 months (rifampin, isoniazid, pyrazinamide, and ethambutol) after the diagnosis of TB, and the TB6 group comprised cured patients treated with intensive-phase treatment for 2 months plus continuation phase treatment for 4 months (rifampin and isoniazid). Healthy control inclusion criteria include gender- and age-matched with TB patients. Any subject diagnosed with any respiratory disease, such as COPD, fibrosis, chronic bronchitis, asthma, or lung cancer, was excluded from this study. In addition, patients with extrapulmonary tuberculosis, autoimmune diseases, infectious diseases, any type of cancer, and other diseases were also excluded from our study.

The research was carried out in strict accordance with the declaration of Helsinki and approved by the Ethics Committee of Zhejiang University Medical College, China. All participants signed a written informed consent and gave their permission to use their blood samples for this study. From each participant, 2 mL peripheral blood was drawn under fasting and anticoagulated with EDTA. After centrifugation, the sample was immediately stored in the refrigerator of −80 °C for the subsequent UPLC-MS/MS analysis.

### Plasma pretreatment in the study of lipidomics

The plasma samples were thawed at room temperature, vortexed for 10 s, and centrifuged at 3000 rpm for 5 min at 4 °C. In total, 50 μL sample of each tube was transferred to a new EP tube, mixed with 1 mL of lipid extraction solution, and vortex for 2 min. The EP tube was mixed with 500 μL water after 5 min of sonication and then vortexed for 1 min, followed by centrifugation at 12,000 rpm for 10 min at 4 °C. The resulting supernatants were collected and dried with nitrogen and re-dissolved with 100 μL mobile phase B. The samples were centrifuged at 14,000 rpm for 15 min at 4 °C after vortex oscillation for 1 min, and then analyzed by UPLC-MS/MS.

### UPLC-MS/MS analysis

The lipid metabolites of plasma samples were separated by ultra-high performance liquid chromatography (UPLC, Shim-pack UFLC SHIMADZU CBM A system, MS, QTRAP^®^ 6500+ System). The model of chromatographic separation was Thermo C30 column (2.1 mm × 100 mm, 2.6 μm), and the column temperature was set at 45 °C. Mobile phase was composed of acetonitrile/water (60/40, v/v) containing 0.04% acetic acid and 5 mmol/L ammonium formate (A), and acetonitrile/isopropanol (10/90, v/v) containing 0.04% acetic acid and 5 mmol/L ammonium formate (B).The elution gradient was set as follows: 0 min, 20% B; 3 min, 50% B; 9 min, 75% B; 15 min, 90% B; equilibrium, 50% B. The flow rate was 350 μL/min, and the injection volume was 2 μL.

Initially separated plasma samples were entered into the QTRAP^®^LC-MS/MS system and scanned in a triple quadrupole containing ion trap. The system is equipped with ESI Turbo ion spray port, which can be operated under positive ion and negative ion mode and controlled by Analyst 1.6.3 software. The parameters of the ESI source were set as the following: the temperature of the ion source was set to 550 °C, the ion spray voltage was set to 5500 V in positive ion mode (or −4500 V in negative ion mode), the ion source gas I, the gas II, the curtain gas were set to 55, 60, and 25 psi, respectively, and the collision gas was set to medium. In total, 10 μmol/L and 100 μmol/L polypropylene glycol solution were used for instrument tuning and quality PPG calibration, respectively. QQQ scans were acquired as MRM experiments with collision gas (nitrogen) set to 5 psi. Declustering potential (DP) and collision energy (CE) for individual MRM transitions were done with further DP and CE optimization. A specific set of MRM transitions were monitored for each period according to the metabolites within this period.

### Raw data processing

The mass spectrum data were processed by Software Analyst 1.6.3. The reproducibility of the extraction and detection of lipid metabolites in the positive and negative ion mode was determined by the total ion flow chromatogram of the mixed QC samples. Metabolite structure analysis referred to some existing mass spectrometry public databases, mainly including massbank (http://www.massbank.jp/), knapsack (http://kanaya.naist.jp/knapsack/), HMDB (http://www.hmdb.ca/), and Metlin (http://metlin.scripps.edu/index.php). Based on the in-house database and other public databases, qualitative analysis was carried out according to the retention time of the test substance and the mass to charge ratio of parent-daughter ions. Lipid was classified and named in strict accordance with LIPID MAPS (www.lipidmaps. ORG). The quantitative analysis was completed by multiple reaction monitoring (MRM) of triple quadrupole mass spectrometry. The signal intensity of characteristic ions was obtained in the detector. MultiQuant was used to integrate and calibrate the chromatographic peaks. The peak area of each chromatographic peak represented the relative content of the corresponding substance.

### Statistical analysis

Chi-square test was adopted for the analysis of baseline characteristics of the study population, and the Kruskal–Wallis *H* test was used to determine the differences between groups. The data of lipidomics were normalized using R software (https://www.r-project.org/). MetaboAnalyst 4.0 (http://www.metaboanalyst.ca) was used to analyze the metabolites using the orthogonal partial least squares (OPLS) model. R^2^X (the interpretability of the model for the categorical variable X) was obtained after cross-validation, R^2^Y (the interpretability of the model for the categorical variable Y), and Q^2^ (predictability of the model) were obtained after cross-validation to judge the validity of the model. K-means clustering method was used to screen lipid metabolites with the same change trend in the plasma of TB0, TB2, TB6, and HC groups. Final results were presented with scatter plots, and a trend chart was made by Graphpad Prism 8.0.2 software. The receiver operator characteristic curve (ROC) was drawn by MedCalc (19.0.7) to analyze the AUC, sensitivity, and specificity of the five candidate biomarkers among groups, and fitting binary logistic regression was used to evaluate the diagnostic value of the combined model.

## Supplementary information

Supplementary information

## Data Availability

The datasets used and analyzed during this study are available from the corresponding author upon reasonable request.
