# Supplementary material for: Novel therapeutic evaluation biomarkers of lipid metabolism targets in uncomplicated pulmonary tuberculosis patients
Source: Signal Transduct Target Ther. 2021 Jan 18;6:22. doi: 10.1038/s41392-020-00427-w (PMC7814055; doi:10.1038/s41392-020-00427-w)
Supplement: Supplementary file 1 — Supplementary information [file 41392_2020_427_MOESM1_ESM.docx]

Supplementary Materials for

Novel therapeutic evaluation biomarkers of lipid metabolism targets in uncomplicated pulmonary tuberculosis patients

Jia-Xi Chen^1,4#^, Yu-Shuai Han^1#^, Shan-Qiang Zhang^2^, Zhi-Bin Li^1^, Jing Chen^1^, Wen-Jing Yi^1,2^, Huai Huang^1,2^, Ting-Ting Jiang^2,3^, Ji-Cheng Li^1,2,3*^

*Correspondence to: [lijichen@zju.edu.cn](mailto:lijichen@zju.edu.cn)

**This PDF file includes:**

Figures. S1

Figure. S1

**a**


**Supplementary Fig. S1 Total ionization chromatography(TIC) of mixed samples.** (a)TIC diagram in positive ion mode. (b)TIC diagram in negative ion mode. The ordinate indicates intensity(CPS), the abscissa indicates retention time(min).

**b**

a
